# Comparative Effects of **α**-, **β**-, and **γ**-Carbolines on Platelet Aggregation and Lipid Membranes

**DOI:** 10.1155/2011/151596

**Published:** 2011-08-11

**Authors:** Hironori Tsuchiya

**Affiliations:** Department of Dental Basic Education, Asahi University School of Dentistry, 1851-1 Hozumi, Mizuho, Gifu 501-0296, Japan

## Abstract

Cigarette smoking and alcohol consumption possibly affect platelet functions. To verify the hypothesis that some **α**-, **β**-, and **γ**-carboline components in cigarette smoke and alcoholic beverages may change platelet aggregability, their effects on human platelets were determined by aggregometry together with investigating their membrane effects by turbidimetry. Carbolines inhibited platelet aggregation induced by five agents with the potency being 3-amino-1,4-dimethyl-5*H*-pyrido[4,3-*b*]indole > 3-amino-1-methyl-5*H*-pyrido[4,3-*b*]indole > 1-methyl-9*H*-pyrido[3,4-*b*]indole. The most potent 3-amino-1,4-dimethyl-5*H*-pyrido[4,3-*b*]indole showed 50% aggregation-inhibitory concentrations of 6–172 **μ**M. Both **γ**-carbolines interacted with phosphatidylcholine membranes to lower the lipid phase transition temperature with the potency correlating to the antiplatelet activity, suggesting that the interaction with platelet membranes to increase their fluidity underlies antiplatelet effects. Given their possible concentration and accumulation in platelets, **γ**- and **β**-carbolines would provide cigarette smokers and alcohol drinkers with reduced platelet aggregability, and they may be responsible for the occurrence of hemorrhagic diseases associated with heavy smoking and alcoholics.

## 1. Introduction

Cigarette smoking has the possibility to affect the functions of platelets. Platelet aggregation stimulated by collagen is diminished in smokers compared with nonsmokers, suggesting that habitual smoking reduces platelet aggregability [1]. Smokers are more susceptible to aspirin in collagen- and adenosine 5′-diphosphate- (ADP-) induced platelet aggregation than nonsmokers [2]. Smoking also promotes the platelet inhibition mediated by an antiplatelet prodrug clopidogrel [3]. Cigarette smoking and alcohol drinking are often characterized by concurrent use, and alcohol drinking is generally accompanied by an increase in smoking. Platelet defects are also noted in alcoholics [4]. Alcohol consumption is inversely related to platelet aggregation in response to collagen, epinephrine, and ADP [5], and it reduces platelet aggregability [6, 7]. However, the influences of cigarette smoking and alcohol consumption on platelet aggregability appear to be conflicting, with some studies indicating a decrease and others an increase of aggregation. Platelet aggregation induced by epinephrine and ADP is increased in habitual smokers [8, 9]. The platelets of alcoholics are initially hypoaggregable but become hyperaggragable after the cessation of drinking [10]. ADP-induced platelet aggregation is increased in alcoholics [11].

Cigarette smoke contains a variety of bioactive compounds [9]. Alcoholic beverages are not consumed as pure ethanol. Therefore, some components in cigarette smoke and alcoholic beverage are assumed to influence the properties of platelets. The objective of this study was to determine antiplatelet effects of the assumed compounds and to relate cigarette smoking and alcohol consumption to hypoaggregability. In addition, their effects on lipid membranes were investigated to address one possible mode of antiplatelet action. From the viewpoint of this mechanistic membrane interaction, an opposite phenomenon, hyperaggregability, in smokers and alcoholics was also discussed.

Cigarette smoking and alcohol drinking have been referred to as risk factors for intracerebral hemorrhage, subarachnoid hemorrhage, and hemorrhagic stroke [12–16]. These factors correlate with each other. Reduced platelet aggregability is involved in the development of hemorrhagic complications [17, 18]. The occurrence of hemorrhage is increased by heavy cigarette smoking and binge alcohol drinking [12, 19]. A graded increase in risk of intracerebral hemorrhage and hemorrhagic stroke depends on how many cigarettes are smoked [13], and several chemicals in smoke are likely to relate to the increased risk of intracranial hemorrhage [20]. Heavy alcohol drinking, but not light-to-moderate alcohol intake, increases the risk of intracerebral and subarachnoid hemorrhage [12, 14]. These suggest that certain antiplatelet components in cigarette smoke and alcoholic beverages are possibly associated with serious hemorrhagic diseases. This study aimed at discussing such a possibility by determining their effects on human platelets.

A class of compounds with the pyridoindole structure, *α*-, *β*-, and *γ*-carbolines, are contained in cigarette smoke and alcoholic beverages. Their levels in mainstream smoke condensates are 2.01–10.3 ng/cigarette for *α*-carboline, 0.25–2.53 *μ*g/cigarette for *β*-carbolines, and 0.29–1.10 ng/cigarette for *γ*-carbolines [21, 22]. The concentrations of *α*- and *γ*-carbolines in combustion smoke samples are 0–1.96 and 0.33–0.74 ng/g in mainstream smoke, respectively, but 5.00–6.51 and 0.27–0.37 ng/g in sidestream smoke, respectively [23]. *β*-carbolines show higher levels of 2.10–8.99 *μ*g/cigarette in sidestream smoke compared with 0.36–4.24 *μ*g/cigarette in mainstream smoke [24]. Various alcoholic beverages contain nM–*μ*M levels of *β*-carbolines depending on brands [25]. Therefore, it is reasonable to assume that the in vivo concentrations of carboline compounds may be increased by smoking cigarettes and consuming alcoholic beverages. The basal concentrations of one of *β*-carbolines, harmane, in plasma (47.7 ± 41.2 pM) and platelets (0.060 ± 0.108 pmol/10^9^  platelets) of smokers are respectively, two- and four-times higher compared with those (22.5 ± 14.3 pM and 0.015 ± 0.008 pmol/10^9^  platelets) of nonsmokers, and harmane concentrations increase to 150.4 ± 110.8 pM and 0.142 ± 0.324 pmol/10^9^  platelets after smoking [26, 27]. Another *β*-carboline, norharmane, also shows plasma and platelet concentrations (114.1 ± 115.3 pM and 0.176 ± 0.078 pmol/10^9^  platelets) in smokers higher than those (56.5 ± 29.7 pM and 0.043 ± 0.027 pmol/10^9^  platelets) in nonsmokers, and norharmane concentrations increase to 1.06 ± 0.52 nM and 0.245 ± 0.086 pmol/10^9^  platelets after smoking [26]. Different *γ*-carbolines are contained in plasma with the concentrations of 18.8 ± 5.0 pM  to  68.3 ± 24.0 pM, which would markedly increase by smoking cigarettes [28, 29]. The plasma levels of norharmane are higher in alcoholics (591.5 ± 154.6 pM) compared with a control group (159.9 ± 63.6 pM) [25]. Harmane in plasma is increased following alcohol ingestion [30]. These elevated concentrations appear to result from inhaling cigarette smoke and drinking alcoholic beverages [31, 32].  

*α*-, *β*-, and *γ*-carbolines exhibit a wide range of bioactivities such as neuropsychiatric, hallucinogenic, and carcinogenic, which have been exclusively studied [33–35]. In addition to these known bioactivities, *α*-, *β*-, and *γ*-carbolines may exert some effects at the periphery, because they are found in human blood and platelets. Certain carbolines such as harmane, harmine, and harmol were recently reported to show the inhibitory effects on collagen-induced platelet aggregation [36]. The structure and antiplatelet activity relationship suggested that the presence of a methyl group in the pyrido moiety is important for carboline compounds to inhibit platelet aggregation [37].

**Figure 1:**
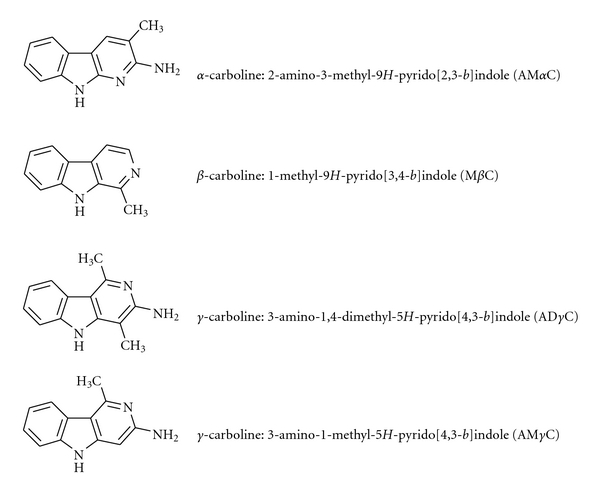
The structures of *α*-, *β*-, and *γ*-carbolines tested in this study.

In this study, the hypothesis that *α*-, *β*-, and *γ*-carbolines may affect the functions of human platelets was verified. 2-Amino-3-methyl-9*H*-pyrido[2,3-*b*]indole (AM*α*C), 1-methyl-9*H*-pyrido[3,4-*b*]indole (M*β*C), 3-amino-1,4-dimethyl-5*H*-pyrido[4,3-*b*]indole (AD*γ*C), and 3-amino-1-methyl-5*H*-pyrido[4,3-*b*]indole (AM*γ*C) (see Figure 1 for their structures) were chosen as tested compounds because they have a methyl substituent as the antiplatelet activity determinant in the common pyrido structure and their concentrations in cigarette smoke and/or alcoholic beverages are relatively high [21–25]. Their effects on platelet aggregation induced by collagen, epinephrine, platelet-activating factor (PAF), ADP, and thrombin were comparatively determined. Consequently, *γ*-carbolines were found to inhibit platelet aggregation in response to all of the tested inducers which have different aggregation mechanisms, suggesting that the site of antiplatelet action of *γ*-carbolines is not confined to receptors or enzymes for individual aggregation agonists. The physicochemical property of biomembranes plays a crucial role in signal transduction and influences the activity of platelets. Changes in membrane fluidity induce the inhibition or the promotion of platelet aggregation stimulated by various aggregants [38–41]. The effects of *γ*-carbolines on lipid membranes were studied to get a clue to one of possible antiplatelet mechanisms.

## 2. Materials and Methods

### 2.1. Chemicals

AM*α*C, M*β*C, AD*γ*C (commercially referred to as Trp-P-1), and AM*γ*C (commercially referred to as Trp-P-2) were purchased from Wako Pure Chemicals (Osaka, Japan). Collagen (MC Medical, Tokyo, Japan), PAF (Funakoshi, Osaka, Japan), epinephrine (Daiichi Sankyo, Tokyo, Japan), ADP (MC Medical) and thrombin (Sigma-Aldrich, St. Louis, Mo, USA) were used for inducing platelet aggregation. 1,2-Dipalmitoylphosphatidylcholine (DPPC) was obtained from Avanti Polar Lipids (Alabaster, Ala, USA). Dimethyl sulfoxide (DMSO) of spectroscopic grade (Kishida, Osaka, Japan) and water of liquid chromatographic grade (Kishida) were used for preparing sample solutions. All other reagents were of the highest grade available.

### 2.2. Platelet Aggregation Assay

The experiments were designed and performed according to the guidelines of the Japanese Pharmacological Society. Human platelet-rich plasma (PRP) and platelet-poor plasma (PPP) were prepared with citrated blood which was obtained from healthy male donors aged 38–48 years (*n* = 6) of nonsmokers and nonalcoholics without taking any drugs at least for one month according to the previous method [42]. Only male subjects were employed as blood donors because platelet aggregation induced by collagen, PAF and ADP was different between males and females in the study which evaluated the comparative effects of the smoking habit and the act of smoking on platelet aggregability [43]. Sharp increases of the carboline concentrations in plasma and platelets were found in nonsmokers after 5 minutes following smoking one cigarette [25–27] and in nonalcoholics 30 minutes after drinking alcoholic beverages [32]. Therefore, PRP and PPP were prepared from nonsmokers and nonalcoholics in order to determine the inherent effects of *α*-, *β*-, and *γ*-carbolines on platelet aggregation without exposing platelets to carboline compounds. The platelet count of PRP was adjusted to be 3 × 10^5^ platelets/*μ*L by diluting PRP with PPP. Both PRP and PPP were used within 3 hours after preparation.

Platelet aggregation was assayed by the same method as the aggregometry using an antiplatelet reference aspirin which was recently reported from our laboratory [37]. Since its methodological validity had been confirmed [44, 45], this study was performed without using references and the obtained results were discussed with referring to our recent report [37]. The aggregation response of PRP was monitored using a HEMA Tracer 601 aggregometer (Niko Bioscience, Tokyo, Japan) by an increase of percent light transmission (% *T*) at 660 nm as a function of time. PPP and unstimulated PRP were defined as 100% *T* and 0% *T*, respectively. The tested carbolines were dissolved in and diluted with DMSO and/or water. After adjustment of  % *T* to 0, 20 *μ*L of the sample solutions were added to 170 *μ*L of PRP. Vehicle alone was added for controls. The volume of DMSO was kept less than 0.1%–1.0% (v/v) of the total volume so as not to affect platelet aggregability and aggregation inducers. After treating PRP with each carboline for 1 minute, platelet aggregation was induced by adding 10 *μ*L of the aqueous solutions of collagen (50 *μ*g/mL), PAF (5 *μ*M), epinephrine (40 *μ*g/mL), ADP (60 *μ*M) or thrombin (5 units/mL). The time of their addition was defined as 0 minute. A maximal  % *T* of aggregation response (*T*_max_), an area under curve of aggregation response (AUC, from 0 to 5 minutes) and a single slope of aggregation response were measured in platelet aggregation induced by collagen, PAF, ADP, and thrombin. Since epinephrine showed the biphasic aggregation, first *T*_max_ (at 1 minute) and second *T*_max_ (at 5 minutes), first AUC (from 0 to 1.5 minutes) and second AUC (from 1.5 to 5 minutes), and slopes of the first (%  *T* at 45 seconds) and second (%  *T* at 4 minutes) phases were measured. The tested compounds were assayed at various concentrations (1–1000 *μ*M). Their  % inhibitions were plotted against their concentrations to prepare concentration-inhibition curves, from which 50% inhibitory concentrations were calculated [46].

### 2.3. Membrane Interaction Assay

The changes in turbidity of liposomal membrane suspensions reflect the thermotropic phase transition of membrane-constituting phospholipids from the gel to the liquid crystalline phase and the phase transition temperatures are altered by membrane-interacting compounds [47]. The membrane interactions of antiplatelet AD*γ*C and AM*γ*C were assayed as reported previously [48]. Briefly, DPPC liposomes were prepared by hydrating the dry film of DPPC (final concentration of 0.4 mM) with water, and then treated with AD*γ*C and AM*γ*C (0, 0.5, 1 and 1.5 mM for each). The absorption of DPPC liposome suspensions was measured at 450 nm by a UV-260 spectrophotometer equipped with a CPS thermocontroller (Shimadzu, Kyoto, Japan) as the temperature was increased. The transition between gel and liquid crystalline phases in phospholipid liposomes dispersed in the aqueous medium is accompanied by a sudden change in turbidity [49]. The phase transition temperatures defined as the mid-point of the abrupt step in absorbance were calculated.

### 2.4. Statistical Analysis

All results are expressed as mean ± SE (*n* = 7 for antiplatelet experiments and *n* = 6 for membrane experiments). Data were analyzed by ANOVA, followed by post hoc Fisher's PLSD test using StatView version 5.0 (SAS Institute, Cary, NC, USA). Values of *P* < .05 were considered statistically significant.

## 3. Results

### 3.1. Antiaggregatory Effect

**Figure 2:**
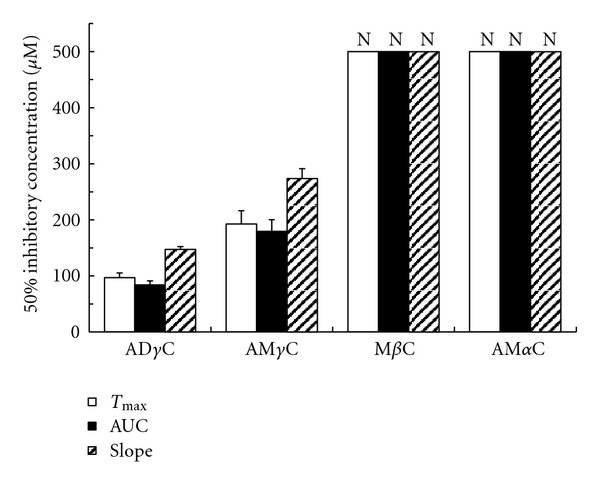
Concentrations of carbolines to produce 50% inhibition of the platelet aggregation responses (*T*_max_, AUC, and slope) to collagen. N: Not inhibited by 50% at the indicated concentrations. Data are presented as mean ± SE (*n* = 7).

**Figure 3:**
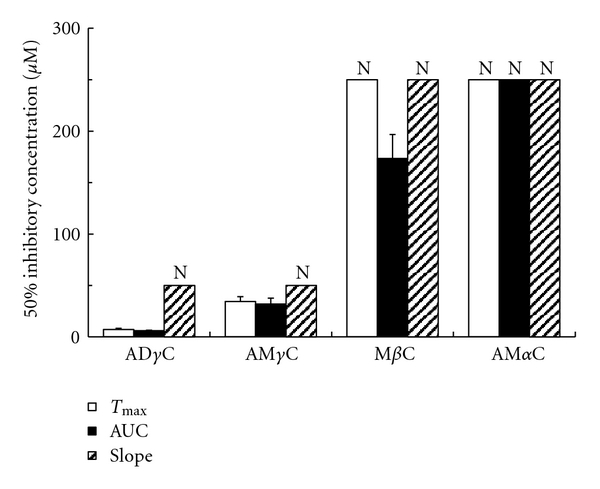
Concentrations of carbolines to produce 50% inhibition of the platelet aggregation responses (*T*_max_, AUC, and slope) to PAF. N: Not inhibited by 50% at the indicated concentrations. Data are presented as mean ± SE (*n* = 7).

**Figure 4:**
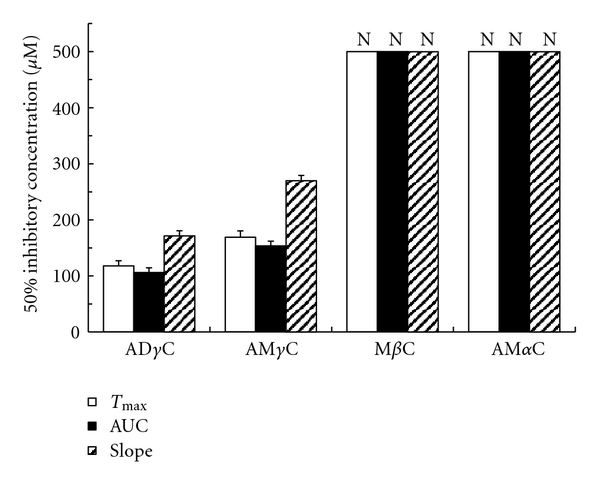
Concentrations of carbolines to produce 50% inhibition of the first phase platelet aggregation responses (*T*_max_, AUC, and slope) to epinephrine. N: Not inhibited by 50% at the indicated concentrations. Data are presented as mean ± SE (*n* = 7).

**Figure 5:**
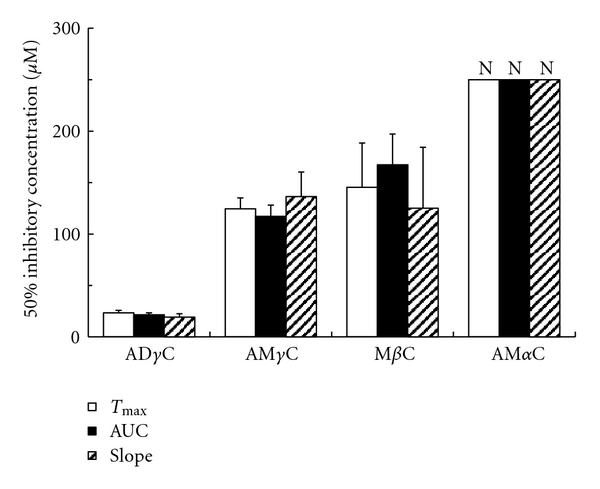
Concentrations of carbolines to produce 50% inhibition of the second phase platelet aggregation responses (*T*_max_, AUC, and slope) to epinephrine. N: Not inhibited by 50% at the indicated concentrations. Data are presented as mean ± SE (*n* = 7).

**Figure 6:**
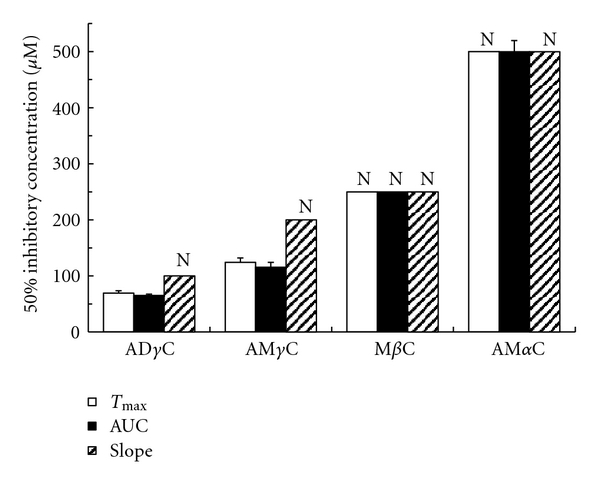
Concentrations of carbolines to produce 50% inhibition of the platelet aggregation responses (*T*_max_, AUC, and slope) to ADP. N: Not inhibited by 50% at the indicated concentrations. Data are presented as mean ± SE (*n* = 7).

**Figure 7:**
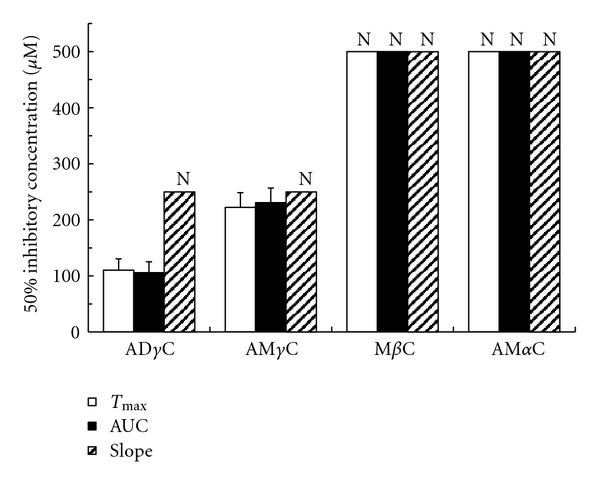
Concentrations of carbolines to produce 50% inhibition of the platelet aggregation responses (*T*_max_, AUC, and slope) to thrombin. N: Not inhibited by 50% at the indicated concentrations. Data are presented as mean ± SE (*n* = 7).

Carboline compounds affected human platelets with the potency different between subclasses. Their inhibitory effects on platelet aggregation induced by collagen, PAF, epinephrine, ADP and thrombin are shown in Figures 2, 3, 4, 5, 6, and 7. The comparisons showed that *γ*-carbolines were the most effective in inhibiting platelet aggregation, followed by less effective *β*-carboline, with *α*-carboline being almost ineffective at the tested concentrations (250 or 500 *μ*M). AM*α*C showed a 50% inhibitory concentration of 499.6 ± 20.3 *μ*M only for AUC of aggregation response to ADP. In contrast, AD*γ*C and AM*γ*C decreased *T*_max_ and AUC of aggregation responses to all of collagen, PAF, epinephrine, ADP, and thrombin. Both *γ*-carbolines also decreased the slopes of collagen- and epinephrine-induced platelet aggregation. When comparing 50% inhibitory concentrations, AD*γ*C was more potent than AM*γ*C. Even at 10 *μ*M or less, AD*γ*C inhibitorily influenced the aggregability of platelets stimulated by PAF and epinephrine.

### 3.2. Membrane Interaction

**Figure 8:**
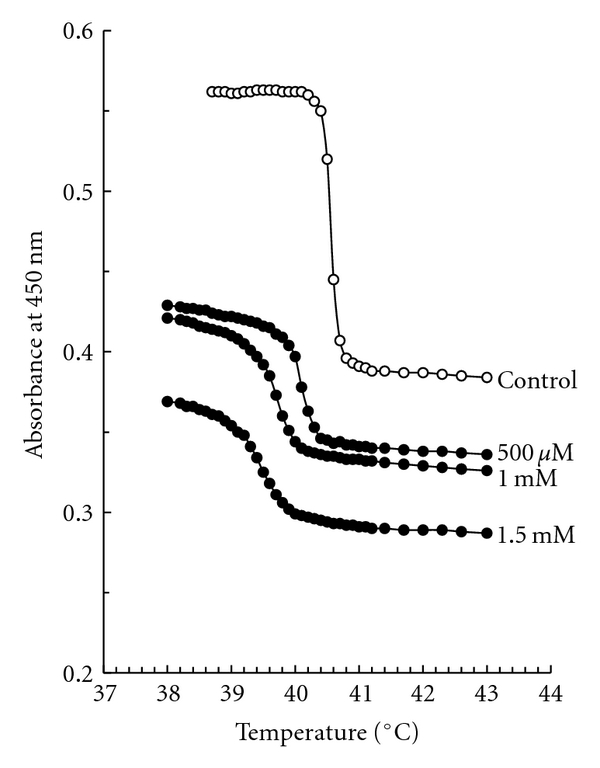
Effects of antiplatelet *γ*-carboline on membrane DPPC phase transition. The absorbance of DPPC liposome suspensions treated with 0, 0.5, 1, or 1.5 mM AD*γ*C was measured at 450 nm with increasing the temperature. Typical traces in multiple measurements are shown.

Antiplatelet *γ*-carbolines influenced the phase transition of membrane DPPC as shown in Figure 8. AD*γ*C lowered the phase transition temperature from a control value of 40.51 ± 0.02°C to 40.07 ± 0.03°C at 500 *μ*M, 39.73 ± 0.03°C at 1 mM and 39.45 ± 0.02°C at 1.5 mM. AM*γ*C shifted to 40.17 ± 0.03°C, 39.93 ± 0.03°C and 39.73 ± 0.02°C at 500 *μ*M, 1 mM and 1.5 mM, respectively. AD*γ*C was more effective in interacting with DPPC membranes than AM*γ*C (*P* < .05 at 500 *μ*M and *P* < .01 at 1 and 1.5 mM).

## 4. Discussion

The comparative studies have revealed that *β*- and *γ*-carbolines, but not *α*-carboline, inhibit platelet aggregation induced by five different agents with the potency being AD*γ*C > AM*γ*C > M*β*C. The relation between structure and antiplatelet activity indicates that the basic structure of 5*H*-pyrido[4,3-*b*]indole (*γ*-carboline) is important for inhibiting platelet aggregation, followed by the structure of 9*H*-pyrido[3,4-*b*]indole (*β*-carboline). An additional methyl group in the pyrido moiety provides *γ*-carbolines with higher activity. Since AD*γ*C and AM*γ*C themselves do not aggregate platelets despite partly resembling serotonin in structure [50], these *γ*-carbolines are referred to as potent antiplatelet compounds.

Antiplatelet *γ*-carbolines showed relatively low concentrations to produce 50% inhibition of AUC of aggregation response. Since the antiplatelet effects of aspirin were recently reported by using the same method for platelet aggregation assay from our laboratory [37], its 50% AUC-inhibitory concentrations are usable for comparing with the activity of *γ*-carbolines. Aspirin showed 116.1 ± 6.4 *μ*M for collagen-induced, 71.3 ± 5.5 *μ*M for epinephrine-induced first phase and 19.3 ± 3.6 *μ*M for epinephrine-induced second phase response, although it did not inhibit PAF-, ADP-, and thrombin-induced responses by 50% at 500 *μ*M. In this study, AD*γ*C and AM*γ*C have been found to show 50% AUC-inhibitory concentrations of 84.0 ± 7.1 and 179.5 ± 20.7 *μ*M, 106.1 ± 8.5 and 153.6 ± 8.5 *μ*M, and 21.5 ± 1.9 and 117.2 ± 11.0 *μ*M for collagen-induced, epinephrine-induced first phase and epinephrine-induced second phase response, respectively. AD*γ*C and AM*γ*C are also effective in inhibiting AUC of aggregation responses induced by PAF, ADP and thrombin to show 50% inhibitory concentrations of 6.01 ± 0.58 and 32.0 ± 5.8 *μ*M, 65.2 ± 2.4 and 115.5 ± 8.8 *μ*M, and 105.8 ± 19.5 and 230.9 ± 25.8 *μ*M, respectively. With respect to platelet aggregation inhibition, *γ*-carbolines, especially AD*γ*C, are comparable to or more potent than aspirin.

A question arises as to whether carboline components actually affect the aggregability of platelets of cigarette smokers and alcohol drinkers. *α*-, *β*-, and *γ*-carbolines are contained in mainstream cigarette smoke (≥22 ng/cigarette for AM*α*C, ≥2.2 *μ*g/cigarette for M*β*C, ≥0.5 ng/cigarette for AD*γ*C and ≥1.1 ng/cigarette for AM*γ*C), and higher levels are found in sidestream cigarette smoke (≥3.0 *μ*g/cigarette for M*β*C) [21, 22, 24, 25]. *β*-carbolines are also present in various alcoholic beverages at the concentrations of ≥3.2 *μ*M for M*β*C [25, 51]. Compared with nonsmokers (≥23 pM), the plasma levels of M*β*C are higher in smokers (≥48 pM) and increase to ≥165 pM by smoking a cigarette [25]. The platelet concentrations of M*β*C are ≥0.015 pmol/10^9^ platelets for nonsmokers but ≥0.060 pmol/10^9^ platelets for smokers, and M*β*C shows the increasing platelet concentrations to be ≥0.142 pmol/10^9^ platelets 13 minutes after smoking [26]. *β*-carbolines increase in blood rapidly following cigarette smoking and alcohol drinking, and their concentrations in platelets are much higher than in plasma, indicating their significant concentration and accumulation in platelets. The accurate *γ*-carboline concentrations in platelets of smokers and alcoholics have been unknown. In the dosing experiment using rabbits, however, AD*γ*C was present in blood, especially in red blood cells, for a long time after oral dosing [52]. Although aspirin is less potent in aggregation inhibition than AD*γ*C, it has been frequently used as an antiplatelet drug. Considering that cigarette smoke- and alcoholic beverage-derived *β*- and *γ*-carbolines are concentrated and accumulated in platelets, the possibility for them to affect platelet aggregability is not necessarily excluded.

Although the platelet aggregation mechanisms for collagen, PAF, epinephrine, ADP, and thrombin differ, AD*γ*C and AM*γ*C were inhibitory on platelet aggregation induced by all of these aggregants, and M*β*C by two aggregants. These results indicate that *γ*- and *β*-carbolines influence the step common to different aggregation agonists in addition to their specific action at the receptor and enzyme levels. The physicochemical property of biomembranes, such as fluidity, plays a crucial role in signal transduction and affects the activity of platelets. Membrane fluidity modulates platelet aggregability and membrane-fluidizing compounds attenuate collagen- and thrombin-induced platelet aggregation [53]. A change in platelet membrane fluidity is mechanistically related to various antiplatelet compounds [38, 54].

The membrane effects of toxins and drugs have been most frequently studied by measuring fluorescence polarization of liposomal and cellular membranes labeled with fluorescent probes [37–39, 54]. However, such a method was not applicable to antiplatelet *γ*-carbolines, because they are naturally fluorescent with the maximal excitation and emission wavelengths almost similar to those of typical fluorescent probes. Therefore, turbidimetry was used for them. The changes in turbidity of liposome suspensions reflect the promotion of a gel to liquid crystalline transition of membrane phospholipids, indicating an increase of membrane fluidity [47, 48]. Consequently, AD*γ*C and AM*γ*C were found to lower the phase transition temperature of membrane DPPC at 500 *μ*M–1.5 mM with the potency correlating to their relative antiplatelet effects. Antiplatelet *γ*-carbolines appear to interact with lipid membranes and increase their fluidity at platelet aggregation-inhibitory concentrations.

The antiplatelet mechanisms previously reported for *β*- and *γ*-carbolines and their relating structures include the inhibition of aggregation-relevant enzymes and receptors, and the suppression of cytosolic calcium mobilization and arachidonic acid liberation. *β*- and *γ*-carbolines influence cyclooxygenase activity and arachidonic acid metabolism to reduce the production of prostaglandins and thromboxane [55]. Antiplatelet *γ*-carbolines (AD*γ*C and AM*γ*C) and *β*-carbolines (M*β*C) are the potent inhibitors of monoamine oxidase and serotonin uptake of platelets [33, 50]. *β*-carbolines like M*β*C inhibit phospholipase C*γ*2 and protein tyrosine phosphorylation [36]. Biological membranes require the lipid bilayer environments optimal for membrane-embedded enzymes, receptors, and transporter systems. The fluidity changes of platelet membranes modify the activities of phospholipase, cyclooxygenase, and aggregation agonists' receptors with the subsequent inhibition of phosphoinositide breakdown, and of prostaglandin and thromboxane formation [38, 39]. The increased membrane fluidity also implies that aggregation-relevant receptors on platelet membranes are less exposed to the external environment.

The present results suggest that platelet hypoaggregability might be induced in smokers and alcoholics by *γ*- and *β*-carbolines. However, the influences of cigarette smoking and alcohol consumption on platelet aggregation have been conflicting, with some studies reporting a reduction of aggregability [1–7] but others an enhancement of aggregability [8–11]. In contrast to fluid membranes (with increased fluidity) induced by AD*γ*C and AM*γ*C, rigid membranes (with decreased fluidity) show the enhanced platelet aggregability in response to epinephrine and thrombin [40, 41]. The decreased membrane fluidity renders platelet receptors more exposed to the external environment and makes the binding of agonists to the receptors more efficiently, resulting in an increase of platelet sensitivity to aggregants (hyperaggregability) [56, 57]. Varying lipid compositions modify the fluidity of biomembranes and cholesterol is one of determinants for decreasing membrane fluidity. While the enhanced platelet aggregability could be found in chronic smokers, subjects who had smoked 10 ± 2 cigarettes per day for 7–10 years showed the decreased fluidity of platelets, which was due to an increase of cholesterol in platelet membranes [58]. Platelet aggregability in response to ADP and collagen was enhanced in alcoholics with increasing cholesterol in platelet membranes [11]. The conflicting phenomena, hypoaggregability and hyperaggregability, associated with cigarette smoking and alcohol consumption appear to be explained by the biphasic effects of membrane fluidity changes.

Reduced platelet aggregability is important in the development of intracranial hemorrhage [17, 28]. Antiplatelet medication is known to increase the incidence of intracerebral hemorrhage, hemorrhagic stroke and other hemorrhagic complication [59, 60] and also increase the recurrence risk of intracerebral hemorrhage [61]. Intracerebral hemorrhage, subarachnoid hemorrhage, and hemorrhagic stroke are closely associated with smoking and drinking habits [12–16]. The present results suggest the possibility that *γ*- and *β*-carboline components in cigarette smoke and/or alcoholic beverages are pharmacotoxicologically relevant to these serious hemorrhagic diseases as well as antiplatelet agents by decreasing platelet aggregation responses to different aggregants.

Another etiological role of cigarette smoking and alcohol consumption has been indicated in cardiovascular events [62, 63] although it is conflicting similarly to their influence on platelet aggregability. Platelet aggregation is pathologically related to coronary artery disease and coronary thrombosis leads to myocardial infarction. The antiplatelet effects of *γ*- and *β*-carbolines are in line with several studies that cigarette smoking and alcohol drinking protect against coronary heart disease and also lower the risk of coronary artery disease and myocardial infarction [63–65]. However, such effects are inconsistent with other studies that cigarette smoking is a risk factor for coronary artery disease and binge alcohol drinking precipitates fatal myocardial infarction [62, 64]. Chronic smokers and heavy drinkers show an increase of cholesterol in platelet membranes [6, 58], by which the membrane fluidity of platelets is decreased. The aggregability should be enhanced in such rigid platelet membranes, increasing cardiovascular morbidity.

In conclusion, the reduced platelet aggregability found in cigarette smokers and alcoholic drinkers would be attributable to antiplatelet carboline components in cigarette smoke and alcoholic beverages. *γ*- and *β*-carbolines may be responsible for the occurrence of hemorrhagic diseases associated with heavy smoking and alcoholics by inhibiting platelet aggregation.

## Abbreviations

AD*γ*C: 3-amino-1,4-dimethyl-5*H*-pyrido[4,3-*b*]indole
ADP: adenosine 5′-diphosphate
AM*α*C: 2-amino-3-methyl-9*H*-pyrido[2,3-*b*]indole
AM*γ*C: 3-amino-1-methyl-5*H*-pyrido[4,3-*b*]indole
AUC: area under curve of aggregation response
DMSO: dimethyl sulfoxide
DPPC: 1,2-dipalmitoylphosphatidylcholine
M*β*C: 1-methyl-9*H*-pyrido[3,4-*b*]indole
PAF: platelet-activating factor
PPP: platelet-poor plasma
PRP: platelet-rich plasma
% *T*: percent light transmission
*T*_max _: maximal % *T* of aggregation response.

## References

[1] Foo LC, Roshidah I, Aimy MB (1991). Platelets of habitual smokers have reduced susceptibility to aggregating agent. *Thrombosis and Haemostasis*.

[2] Weber AA, Liesener S, Schanz A, Hohlfeld T, Schrör K (2000). Habitual smoking causes an abnormality in platelet thromboxane A2 metabolism and results in an altered susceptibility to aspirin effects. *Platelets*.

[3] Gremmel T, Steiner S, Seidinger D, Koppensteiner R, Panzer S, Kopp CW (2009). Smoking promotes clopidogrel-mediated platelet inhibition in patients receiving dual antiplatelet therapy. *Thrombosis Research*.

[4] Rubin R (1999). Effect of ethanol on platelet function. *Alcoholism: Clinical and Experimental Research*.

[5] Mukamal KJ, Massaro JM, Ault KA (2005). Alcohol consumption and platelet activation and aggregation among women and men: the Framingham Offspring Study. *Alcoholism: Clinical and Experimental Research*.

[6] Renaud SC, Ruf JC (1996). Effects of alcohol on platelet functions. *Clinica Chimica Acta*.

[7] Zhang QH, Das K, Siddiqui S, Myers AK (2000). Effects of acute, moderate ethanol consumption on human platelet aggregation in platelet-rich plasma and whole blood. *Alcoholism: Clinical and Experimental Research*.

[8] Fusegawa Y, Handa S (2000). Platelet aggregation induced by ADP or epinephrine is enhanced in habitual smokers. *Thrombosis Research*.

[9] Ambrose JA, Barua RS (2004). The pathophysiology of cigarette smoking and cardiovascular disease: an update. *Journal of the American College of Cardiology*.

[10] Desai K, Owen JS, Wilson DT, Hutton RA (1986). Platelet aggregation and plasma lipoproteins in alcoholics during alcohol withdrawal. *Thrombosis and Haemostasis*.

[11] Watanabe M, Shiraishi K, Itakura M, Matsuzaki S (1998). Relationship between platelet membrane lipid compositions and platelet aggregability in alcoholic liver disease. *Alcoholism: Clinical and Experimental Research*.

[12] Longstreth WT, Nelson LM, Koepsell TD, van Belle G (1992). Cigarette smoking, alcohol use, and subarachnoid hemorrhage. *Stroke*.

[13] Kurth T, Kase CS, Berger K, Schaeffner ES, Buring JE, Gaziano JM (2003). Smoking and the risk of hemorrhagic stroke in men. *Stroke*.

[14] Ariesen MJ, Claus SP, Rinkel GJE, Algra A (2003). Risk factors for intracerebral hemorrhage in the general population: a systematic review. *Stroke*.

[15] Juvela S, Hillbom M, Palomäki H (1995). Risk factors for spontaneous intracerebral hemorrhage. *Stroke*.

[16] Kurth T, Kase CS, Berger K, Gaziano JM, Cook NR, Buring JE (2003). Smoking and risk of hemorrhagic stroke in women. *Stroke*.

[17] Neiman J, Rand ML, Jakowec DM, Packham MA (1989). Platelet responses to platelet-activating factor are inhibited in alcoholics undergoing alcohol withdrawal. *Thrombosis Research*.

[18] Juvela S, Kaste M (1991). Reduced platelet aggregability and thromboxane release after rebleeding in patients with subarachnoid hemorrhage. *Journal of Neurosurgery*.

[19] Juvela S, Hillbom M, Numminen H, Koskinen P (1993). Cigarette smoking and alcohol consumption as risk factors for aneurysmal subarachnoid hemorrhage. *Stroke*.

[20] Koskinen LOD, Blomstedt PC (2006). Smoking and non-smoking tobacco as risk factors in subarachnoid haemorrhage. *Acta Neurologica Scandinavica*.

[21] Manabe S, Wada O, Kanai Y (1990). Simultaneous determination of amino-*α*-carbolines and amino-*γ*-carbolines in cigarette smoke condensate by high-performance liquid chromatography. *Journal of Chromatography—Biomedical Applications*.

[22] Smith CJ, Qian X, Zha Q, Moldoveanu SC (2004). Analysis of *α*- and *β*-carbolines in mainstream smoke of reference cigarettes by gas chromatography-mass spectrometry. *Journal of Chromatography A*.

[23] Kataoka H, Kijima K, Maruo G (1998). Determination of mutagenic heterocyclic amines in combustion smoke samples. *Bulletin of Environmental Contamination and Toxicology*.

[24] Totsuka Y, Ushiyama H, Ishihara J (1999). Quantification of the co-mutagenic *β*-carbolines, norharman and harman, in cigarette smoke condensates and cooked foods. *Cancer Letters*.

[25] Pfau W, Skog K (2004). Exposure to *β*-carbolines norharman and harman. *Journal of Chromatography B*.

[26] Rommelspacher H, Meier-Henco M, Smolka M, Kloft C (2002). The levels of norharman are high enough after smoking to affect monoamineoxidase B in platelets. *European Journal of Pharmacology*.

[27] Talhout R, Opperhuizen A, van Amsterdam JGC (2007). Role of acetaldehyde in tobacco smoke addiction. *European Neuropsychopharmacology*.

[28] Manabe S, Wada O (1988). Analysis of human plasma as an exposure level monitor for carcinogenic tryptophan pyrolysis products. *Mutation Research*.

[29] Manabe S, Wada O (1990). Carcinogenic tryptophan pyrolysis products in cigarette smoke condensate and cigarette smoke-polluted indoor air. *Environmental Pollution*.

[30] Rommelspacher H, Damm H, Lutter S (1990). Harman (1-methyl-*β*-carboline) in blood plasma and erythrocytes of nonalcoholics following ethanol loading. *Alcohol*.

[31] Spijkerman R, van den Eijnden R, van de Mheen D, Bongers I, Fekkes D (2002). The impact of smoking and drinking on plasma levels of norharman. *European Neuropsychopharmacology*.

[32] Tsuchiya H, Yamada K, Tajima K, Hayashi T (1996). Urinary excretion of tetrahydro-*β*-carbolines relating to ingestion of alcoholic beverages. *Alcohol and Alcoholism*.

[33] Herraiz T, González D, Ancín-Azpilicueta C, Arán VJ, Guillén H (2010). *β*-Carboline alkaloids in *Peganum harmala* and inhibition of human monoamine oxidase (MAO). *Food and Chemical Toxicology*.

[34] Grella B, Dukat M, Young R (1998). Investigation of hallucinogenic and related *β*-carbolines. *Drug and Alcohol Dependence*.

[35] Pfau W, Marquardt H (2001). Cell transformation in vitro by food-derived heterocyclic amines Trp-P-1, Trp-P-2 and N^2^-OH-PhIP. *Toxicology*.

[36] Im JH, Jin YR, Lee JJ (2009). Antiplatelet activity of *β*-carboline alkaloids from *Perganum harmala*: a possible mechanism through inhibiting PLC*γ*2 phosphorylation. *Vascular Pharmacology*.

[37] Tsuchiya H, Ohmoto S (2010). Comparative effects of *β*-carbolines on platelet aggregation and lipid membranes. *Pharmacological Reports*.

[38] Sheu JR, Hsiao G, Luk HN (2002). Mechanisms involved in the antiplatelet activity of midazolam in human platelets. *Anesthesiology*.

[39] Sheu JR, Lee YM, Lee LW, Luk HN, Yen MH (1998). Inhibitory mechanisms of naloxone on human platelets. *Clinical and Experimental Pharmacology and Physiology*.

[40] Srivastava K, Dash D (2001). Altered membrane fluidity and signal transduction in the platelets from patients of thrombotic stroke. *Molecular and Cellular Biochemistry*.

[41] Srivastava K, Dash D (2002). Changes in membrane microenvironment and signal transduction in platelets from NIDDM patients—a pilot study. *Clinica Chimica Acta*.

[42] Tsuchiya H, Sato M, Watanabe I (1999). Antiplatelet activity of soy sauce as functional seasoning. *Journal of Agricultural and Food Chemistry*.

[43] Taylor RR, Sturm M, Vandongen R, Strophair J, Beilin LJ (1987). Whole blood platelet aggregation is not affected by cigarette smoking but is sex-related. *Clinical and Experimental Pharmacology and Physiology*.

[44] Furusawa M, Tsuchiya H, Nagayama M, Tanaka T, Nakaya K, Iinuma M (2003). Anti-platelet and membrane-rigidifying flavonoids in brownish scale of onion. *Journal of Health Science*.

[45] Tsuchiya H, Tanaka T, Nagayama M, Oyama M, Iinuma M (2008). Membrane activity-guided isolation of antiproliferative and antiplatelet constituent from *Evodiopanax innovans*. *Natural Product Communications*.

[46] Odawara A, Kikkawa K, Katoh M, Toryu H, Shimazaki T, Sasaki Y (1996). Inhibitory effects of TA-993, a new 1,5-benzothiazepine derivative, on platelet aggregation. *Circulation Research*.

[47] Korkmaz F, Severcan F (2005). Effect of progesterone on DPPC membrane: evidence for lateral phase separation and inverse action in lipid dynamics. *Archives of Biochemistry and Biophysics*.

[48] Mizogami M, Tsuchiya H, Ueno T, Kashimata M, Takakura K (2008). Stereospecific interaction of bupivacaine enantiomers with lipid membranes. *Regional Anesthesia and Pain Medicine*.

[49] Ueda I, Tashiro C, Arakawa K (1977). Depression of phase-transition temperature in a model cell membrane by local anesthetics. *Anesthesiology*.

[50] Manabe S, Kanai Y, Ishikawa S, Wada O (1988). Carcinogenic tryptophan pyrolysis products potent inhibitors of type A monoamine oxidase and the platelet response to 5-hydroxytryptamine. *Journal of Clinical Chemistry and Clinical Biochemistry*.

[51] Herraiz T (2000). Analysis of the bioactive alkaloids tetrahydro-*β*-carboline and *β*-carboline in food. *Journal of Chromatography A*.

[52] Manabe S, Kanai Y, Wada O (1989). Exposure level monitor of 3-amino-1,4-dimethyl-5H-pyrido[4,3-b]indole, a dietary carcinogen, in rabbits. *Environmental and Molecular Mutagenesis*.

[53] Vlasic N, Medow MS, Schwarz SM, Pritchard KA, Stemerman MB (1993). Lipid fluidity modulates platelet aggregation and agglutination in vitro. *Life Sciences*.

[54] Chiu HF, Yang SP, Kuo YL, Lai YS, Chou TC (2006). Mechanisms involved in the antiplatelet effect of C-phycocyanin. *British Journal of Nutrition*.

[55] Ishikawa S, Manabe S, Yanagisawa H, Kitagawa Y, Kanai Y, Wada O (1987). Inhibitory effects of tryptophan pyrolysis products on human platelet aggregation through inhibition of prostaglandin endoperoxide synthetase. *Food and Chemical Toxicology*.

[56] Watala C (1991). May the alterations in lipid fluidity-mediated platelet hypersensitivity contribute to accelerated aging of platelets in diabetes mellitus?. *Medical Hypotheses*.

[57] Watala C, Golański J, Walkowiak B (1996). Does reduced membrane lipid fluidity underlie the altered thrombin-induced expression of integrin *α*
_IIb_
*β*
_3_ and PADGEM-140 in membranes of platelets from diabetic juveniles?. *Platelets*.

[58] Padmavathi P, Reddy VD, Maturu P, Varadacharyulu N (2010). Smoking-induced alterations in platelet membrane fluidity and Na^+^/K^+^-ATPase activity in chronic cigarette smokers. *Journal of Atherosclerosis and Thrombosis*.

[59] Björklund L, Wallander MA, Johansson S, Lesén E (2009). Aspirin in cardiology—benefits and risks. *International Journal of Clinical Practice*.

[60] Naidech AM, Bendok BR, Garg RK (2009). Reduced platelet activity is associated with more intraventricular hemorrhage. *Neurosurgery*.

[61] Biffi A, Halpin A, Towfighi A (2010). Aspirin and recurrent intracerebral hemorrhage in cerebral amyloid angiopathy. *Neurology*.

[62] Inoue T (2004). Cigarette smoking as a risk factor on coronary artery diseases and its effects on platelet function. *Tobacco Induced Diseases*.

[63] Mukamal KJ (2006). The effects of smoking and drinking on cardiovascular disease and risk factors. *Alcohol Research and Health*.

[64] Numminen H, Syrjälä M, Benthin G, Kaste M, Hillbom M (2000). The effect of acute ingestion of a large dose of alcohol on the hemostatic system and its circadian variation. *Stroke*.

[65] Jeong YH, Cho JH, Kang MK (2010). Smoking at least 10 cigarettes per day increases platelet inhibition by clopidogrel in patients with ST-segment-elevation myocardial infarction. *Thrombosis Research*.

